# Excess of the endocannabinoid anandamide during lactation induces overweight, fat accumulation and insulin resistance in adult mice

**DOI:** 10.1186/1758-5996-4-35

**Published:** 2012-07-23

**Authors:** Carolina A Aguirre, Valeska A Castillo, Miguel N Llanos

**Affiliations:** 1 Laboratorio de Nutrición y Regulación Metabólica, Instituto de Nutrición y Tecnología de los Alimentos (INTA), Universidad de Chile, Casilla 138-11, El Líbano, 5524 Santiago, Chile

**Keywords:** Anandamide, Overweight, Insulin resistance, Endocannabinoid system, Adipose tissue

## Abstract

**Background:**

Environmental conditions in early life can induce permanent physiological changes, sometimes increasing the risk of chronic diseases during adulthood. Neural and peripheral circuits controlling energy balance may be modulated during such a critical period. Since type 1 cannabinoid receptors (CB_1_R) have recently emerged as targets for modulating energy balance, their premature chronic activation during early life may result in long-term metabolic consequences associated to overweight/obesity. Endogenous activation of CB_1_R mainly occurs after binding to the endocannabinoid Anandamide (AEA).

**Objective:**

To evaluate long-term effects of AEA treatment during lactation on body weight, epididymal fat accumulation and related metabolic parameters during adulthood.

**Design:**

Male mice pups were orally treated with a solution of AEA (20 μg/g body weight in soy oil) or vehicle during the whole lactation period. After weaning, food intake and body weight were recorded every 10 days. Adult animals were subjected to glucose and insulin tolerance tests. Subsequently, animals were sacrificed and epididymal fat pads were extracted. Circulating levels of plasma insulin, leptin, non-sterified fatty acids (NEFA), triglyceride and cholesterol were also evaluated.

**Results:**

AEA-treated mice during lactation showed a significant increase in accumulated food intake, body weight and epididymal fat during adulthood when compared to control mice. When evaluating CB_1_R protein expression in epididymal fat, the AEA-treated group showed a 150 % increase in expression compared to the control mice. This group also displayed significantly higher levels of circulating glucose, insulin, leptin, triglycerides, cholesterol and NEFA. Moreover, a marked state of insulin resistance was an important finding in the AEA-treated group.

**Conclusion:**

This study showed that overweight, accumulation of visceral fat and associated metabolic disturbances, such as a higher lipid profile and insulin resistance, can be programmed by a treatment with the endocannabinoid AEA during lactation in adult mice.

## Background

The increased body of research referring to early life events with long-term consequences, arose from earlier epidemiological studies linking environmental conditions during infancy to a higher risk of disease and mortality in adulthood [1,2]. This situation is a consequence of permanent changes in physiology and/or structure in response to environmental conditions, due to the developmental plasticity of living organisms.

Overweight/obesity is a physiopathological condition characterized by an imbalance between energy intake and energy expenditure, which may have its origin in early stages of life. This energy balance is controlled by complex central and peripheral systems where the endocannabinoid system has been recently recognized as a critical participant involved in modulating energy homeostasis, with a role in obesity development [3].

The endocannabinoid system is mainly composed by type 1 and 2 cannabinoid receptors (CB_1_R, CB_2_R), their endogenous ligands, named endocannabinoids, as well as the enzymes responsible of their synthesis and hydrolysis. The two most studied endocannabinoids, arachidonoylethanolamide (anandamide; AEA) and 2-arachidonoylglycerol (2-AG), are synthesized from membrane phospholipids and are able to activate different pathways depending on a specific target receptor. Type-1 cannabinoid receptors expressed in the central nervous system and in several peripheral tissues including liver, skeletal muscle and adipose tissue, are mainly activated by AEA [4-6]. The CB_2_R, which is mainly activated by 2-AG, is expressed in immune and hematopoietic cells. However, recent studies have reported the presence of CB_2_R in some areas of the central nervous system and in peripheral tissues such as the liver and pancreas [7-9].

Type 1 receptors are involved in the control of food intake and energy homeostasis [10]. Thus, activation of CB_1_R in the hypothalamus increases appetite [11-13], while its activation in peripheral tissues promotes energy storage by food intake-independent mechanisms, stimulating lipid accumulation in adipocytes [14], and *de novo* lipogenesis in the liver [5]. Therefore, hyperactivation of the endocannabinoid system has been proposed to be important in promoting overweight/obesity and its metabolic consequences [15-17]. Moreover, blocking endocannabinoid action with a CB_1_R antagonist such as Rimonabant or AM-251, improves most features of the metabolic syndrome. This suggests that increased endocannabinoid tone may be its unifying pathogenic cause [18].

Higher endocannabinoid levels during lactation could be originated from maternal feeding or in response to both acute and repetitive stress [19]. Anandamide and 2-AG are synthesized from phospholipids containing arachidonic acid (AA), which together with linoleic acid belong to the n-6 family of essential fatty acids. Since AA present in tissues is obtained from the diet, it is expected that a high intake of n-6 polyunsaturated fatty acids could lead to elevated endocannabinoid levels in different tissues. In the mammary gland, this condition may result in a higher content of AEA in maternal milk [20], which in turn should increase availability of this endocannabinoid for lactating pups, with unknown consequences during adult life. Taking the above mentioned into account, the aim of this study was to evaluate whether an excess of AEA during lactation could induce overweight/obesity in adult mice together with a disrupted metabolic profile.

## Materials and methods

All procedures performed in this study were approved by the Bioethics´ Committee for Animal Experimentation of the Institute of Nutrition and Food Technology, University of Chile. Santiago, Chile.

### Animals

Synchronously pregnant female CD-1 mice were kept in the animal house under normal conditions of humidity and temperature (22-24 °C), on a 12:12 h light–dark cycle. Animals had free access to purified tap water and food. A normal diet of 4 Kcal/g, equivalent to 2.8 assimilated Kcal/g (Champion Co, Santiago, Chile), was used during the entire study [21].

From day 16, pregnant female mice were daily examined at 9:00 and 19:00 h for the presence of pups. Twelve to 16 h after pup detection, 6-8 litters of homogeneous size (12-14 pups) were put together and males separated from females. Afterwards, six male pups that exhibited homogeneous weights were randomly selected and assigned to a substitute mother for random cross lactation. Animals were then assigned to one of the following groups:

1) Control mice: during the entire lactation (21 d), pups were removed daily from the home cage, weighed, and 1 μl/g of body weight of soy oil was orally given.

2) AEA-treated mice: during the entire lactation (21 d), pups were removed daily from the home cage, weighed, and 20 μg/g body weight of AEA (Sigma-Aldrich Co, St Luis, MO, USA) in soy oil (1 μl/g body weight) was orally given.

At 21 days of age, animals were separated from their mothers, and groups of three animals were placed in new cages until 150 days of age. During this period, body weight, food intake, basal metabolic rate, glucose tolerance and insulin sensitivity were evaluated. Adult mice were then sacrificed according to the guidelines for rodent euthanasia provided by the American Medical Veterinary Association [22]. After sacrifice, the whole epididimal fat pads were extracted from the abdominal area and weighed. In addition, blood samples were obtained from the abdominal aorta to evaluate circulating levels of several metabolic markers as further described.

### Body weight and food intake

Body weight and food intake were recorded every 10 days. The amount of accumulated food intake per cage (containing three mice) was calculated by subtracting the lost food inside the cage due to spilling from day 21 to 150.

### Basal metabolic rate

Measurements of basal metabolic rate were done in post-absorptive (four hour fasted) and resting 150 day old mice during the inactive phase and within the thermoneutral zone (30 ± 0.5 °C), using standard flow-through respirometry methods [23].

### Western blot of CB_1_R in epididymal fat

For western blotting procedures, epididymal fat from AEA-treated and control animals was homogenized (Heidolph homogenizer DIAX 600) in 500 μl of RIPA buffer (25 mM Tris–HCl pH7.6, 150 mM NaCl, 1 % sodium deoxycholate, 0.1 % SDS) in the presence of protease inhibitor cocktail (Sigma-Aldrich, catalog number P2714). The protein was separated in a 10 % SDS-polyacrylamide gel (Mini protean III System; BIO-RAD) and subsequently transferred to a PVDF membrane during the overnight at 4 °C. A polyclonal antibody for CB_1_R was used as the primary antibody (Cayman Chemical, CA, USA) and an enzyme-conjugated anti-rabbit antibody was used as the secondary antibody (Bio-Rad, CA, USA). CB_1_R was visualized by chemiluminiscence (Western lightning *Plus*-ECL, enhanced chemiluminescence substrate; Perkin Elmer). The obtained protein bands were normalized against β-actin expression and quantified using Gel-Pro Analyzer 3.1 programme.

### Glucose and insulin tolerance tests

Initially, mice were fasted for 6 h and subsequently injected with a glucose solution (1.5 mg/g body weight intraperitoneal). Tail blood was collected immediately before glucose injection and after 15, 30, 60, 90, and 120 min to evaluate blood glucose levels. Intraperitoneal insulin tolerance tests (ITT) were performed on 6 h fasted mice injected with 0.75 U/kg insulin (Eli Lilly) and tail blood glucose levels were measured (basal, and 15, 30, 45 and 60 min after injection). Blood glucose levels were determined with an Accu-Check Performa glucometer (Roche Diagnostics, Mannheim, Germany). The incremental area under the curve (IAUC) between 0 and 120 min for the glucose tolerance test (GTT), and the decremental area under de curve (DAUC) between 0 and 60 min for ITT, were determined using the trapezoidal method [24].

### Plasma glucose, lipids, and hormonal levels

Plasma levels of glucose, triglycerides, and cholesterol were assessed in triplicate using a commercial enzymatic kit (DIALAB, Neudorf, Austria). Plasma leptin levels were assessed in duplicate using a commercial colorimetric sandwich ELISA Kit (R&D System, Minneapolis, MN). A commercial ELISA kit was also used to measure plasma insulin concentration from duplicate samples (Linco Research, St Charles, MO). Nonesterified fatty acids (NEFA) concentration in plasma samples were measured using a HR Series NEFA-HR(2) Kit (Wako Chemicals USA, Richmond, VA).

### Statistical analysis

Data were expressed as mean ± SEM. Shapiro-Wilk´s and Levene tests were previously done to evaluate normal distribution of data and variance homogeneity. The Mann-Withney *U* Test or 1-way ANOVA statistical analyses were performed, when appropriate. Statistical significance was set at *P*≤0.05. All analyses were conducted using Stata 10.1 statistical package [25].

## Results

### Body weight, epididymal Fat and adiposity index

**Figure 1:**
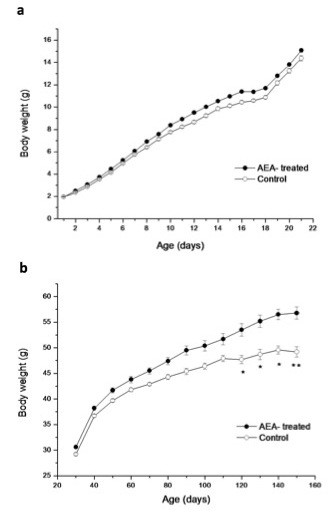
**Body weight time course of AEA-treated mice and control, (a) during lactation and (b) at different ages from days 30 to 150**. (*P < 0.05/**P < 0.01 Mann–Whitney *U* test; mean ± SEM; *n* = 12/group).

**Figure 2:**
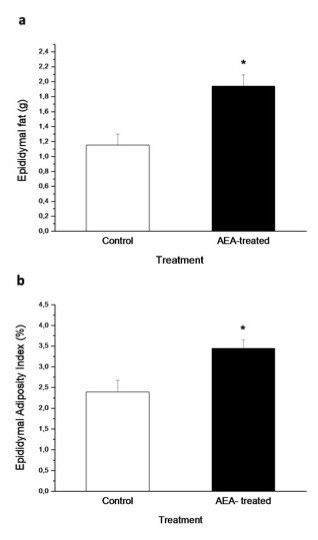
**Effect of AEA treatment during lactation on, (a) the total amount of epididymal fat, and (b) epididymal adiposity Index of adult control and AEA-treated mice**. (*P<0.05; Mann–Whitney *U* Test; mean ± SEM; *n* = 9/group).

Body weight gain through lactation in both groups of mice is shown in Figure 1a. At the end of lactation (day 21) there were no significant differences in body weight between the control and AEA-treated mice. Time course body weight from days 30 to 150 showed that AEA-treated mice had higher body weight, although significant differences to control mice were obtained from day 120 (Figure 1b). This difference was maintained until day 150, where this group reached a body weight 15 % higher than the control mice (55.73 ± 1.3 g *vs* 48.73 ± 1.08 g; *n* = 12; *P* < 0.01). In addition to the increased body weight found in AEA-treated mice, these animals also had a marked increase in epididymal fat content (Figure 2a) when compared to control mice (1.94 ± 0.15 g *vs* 1.15 ± 0.15 g; *n* = 12; *P* < 0.05). This fact accounts for the higher epididymal adiposity index obtained in AEA-treated mice, which is determined as the percentage of the total amount of epididymal fat relative to body weight (Figure 2b).

### Food intake

The accumulated food intake during 130 days (day 21 to 150) in eight cages (three mice per cage) from both groups, shows that AEA-treated mice ate 4.8 % more food than control animals (2033 ± 32 g *vs* 1939 ± 28 g; *P* < 0.05).

### Basal metabolic rate

The basal metabolic rate between both groups showed no significant difference. Thus, oxygen consumption in 150 day-old control and AEA-treated mice was 1.6 ± 0.1 *vs* 1.7 ± 0.1 ml O_2_/g/h, respectively.

### Western blot analysis of CB_1_R in epididymal fat

**Figure 3:**
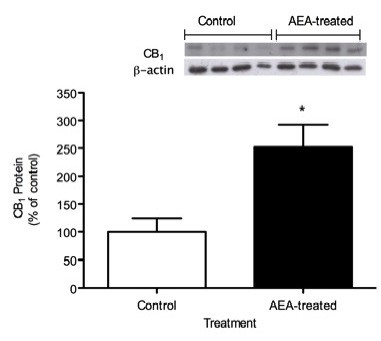
**Relative levels of CB**_**1**_**R protein in epididymal fat of adult AEA-treated and control mice**. For densitometric quantification purposes, β-actin was used as loading control. Results are expressed as percentage relative to the expression in control mice. (* *P* < 0.05; Mann–Whitney *U* test; mean ± SEM; *n* = 6/group).

Figure 3 shows CB_1_R protein expression in epididymal fat of 150 day-old control and AEA-treated mice. It was observed that AEA-treated mice had 150 % higher expression than control mice (*P* < 0.05). Quantification of CB_1_R bands were normalized against β-actin expression.

### Glucose tolerance and insulin tolerance test

**Figure 4:**
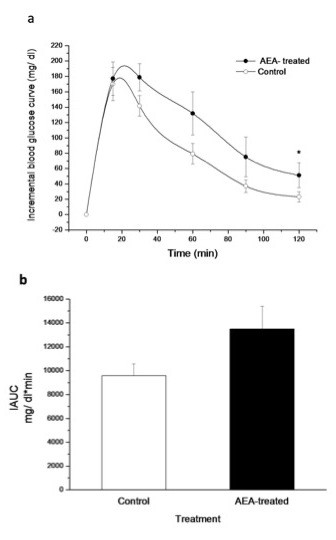
**(a) Increase in blood glucose levels over basal glycemia during GTT performed in adult control and AEA-treated mice and, (b) glucose incremental area under the curve (IAUC) for both groups**. Basal glycemia was measured immediately before intraperitoneal glucose administration (1.5 mg/g of body weight). (*P<0.05; Mann–Whitney *U* Test; mean ± SEM; *n* = 9/group).

**Figure 5:**
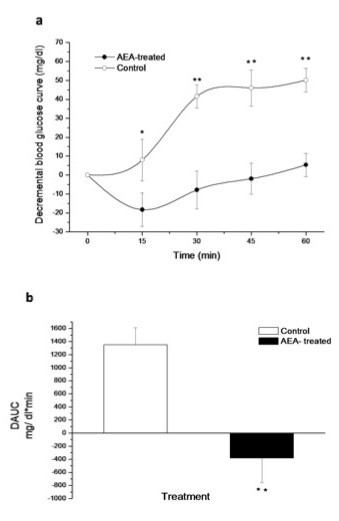
**(a) Decrease in blood glucose levels during ITT in adult control and AEA-treated mice and, (b) decremental glucose area under the curve (DAUC) for both groups**. Basal glycemia was measured immediately before intraperitoneal 0.75 U/Kg insulin administration. (*P < 0.05/**P < 0.01 Mann–Whitney *U* test; mean ± SEM; *n* = 9/group).

The glucose tolerance test performed in 150 day-old mice, only revealed a significantly higher blood glucose level in AEA-treated mice after 120 min (Figure 4a). Incremental AUC was 40 % higher in AEA-treated mice; however, this difference was not significant (Figure 4b). To determine the effect of AEA treatment on insulin sensitivity, an insulin tolerance test was performed. A glucose-lowering effect of insulin was only observed in control mice, which showed higher decrements in blood glucose than AEA-treated mice (Figure 5a). AEA-treated mice have a lower or negative decremental curve, indicating a marked insulin resistance state in these animals and as a result, a lower DAUC (Figure 5b).

### Plasma glucose, lipids and hormonal levels

Overweight induced by AEA during lactation was associated to changes in circulating metabolic markers common to metabolic syndrome. Thus, elevated levels of blood glucose, plasma insulin, leptin and triglycerides were observed. Due to higher epididymal fat accumulation, which may be representative of a higher visceral fat mass, AEA-treated mice showed increased NEFA plasma levels (Table 1).

**Table 1:** Plasma lipid profile, glucose, insulin and leptin levels in 150 day-old, control and AEA-treated mice during lactation

**Plasma levels**	**Control**	**AEA-treated**
TG (mg/dl)	114.4 ± 10.0	144.0 ± 6.0 *
Cholesterol (mg/dl)	100.6 ± 3.8	115.6 ± 3.1 *
NEFA (mg/dl)	2.7 ± 0.6	5.0 ± 0.7*
Glycemia (mg/dl)	114.7 ± 8.6	144.7 ± 7.0 **
Insulin (ng/ml)	1.6 ± 0.2	4.6 ± 0.6 **
Leptin (ng/ml)	5.8 ± 1.3	16.3 ± 2.5 **

Data represent the mean ± SEM of 10 mice from each group. *P < 0.05. **P < 0.01

## Discussion

In this study, we demonstrated that mice treated with an excess of the endocannabinoid anandamide (AEA) during lactation showed increased body weight, epididymal fat accumulation, and a marked insulin resistance state during adulthood. Therefore, the long term pathophysiological condition programmed in such a critical period of life may be a consequence of a transient hyperactivation of CB_1_R. Genetically and diet-induced obese animal models have shown the presence of elevated levels of endocannabinoids in central and peripheral tissues [5,17,26]. Animal studies, together with clinical data in humans, indicate that the impaired energy balance observed in obesity is associated with an overactive endocannabinoid system [16,27].

In the present study, AEA-treated mice ate 4.8 % more food than control mice; however, this could only explain close to half of the observed difference in body weight, suggesting a lower energy expenditure of AEA-treated mice. Basal metabolic rate was remained unchanged between control and AEA-treated mice, indicating a difference in energy expenditure, likely due to decreased locomotor activity in AEA-treated adult mice. This finding is supported by studies where rats chronically treated with a CB_1_R antagonist (rimonabant), showed a transient reduction in food intake but a long lasting reduction in body weight, indicating a metabolic effect independent of energy intake [28]. Moreover, in high fat-fed dogs that normally develop abdominal obesity, chronic treatment with rimonabant reduced the abdominal fat mass. Interestingly, this fact was not related to the transient reduction in food intake and was not associated to any change in basal metabolic rate [29].

Obesity is one of the major components of metabolic syndrome and it is associated to elevated plasma levels of NEFA and triglycerides (TG), which contribute to insulin resistance in peripheral tissues, leading to the development of Type 2 Diabetes [30,31]. In this study, overweight and the higher accumulation of epididymal fat in AEA-treated mice were followed by higher blood glucose levels, elevated plasma levels of TG and a marked insulin resistance state. Altogether, these results characterize main markers of the metabolic syndrome.

Multiple pathways may lead to insulin resistance. Enhanced availability of free fatty acids released from adipose tissue as well as dietary fatty acids have been shown to result in increased amounts of ectopic lipid stores in non-adipose tissues, like skeletal muscle and liver [32]. These lipids, together with their metabolites, are able to contribute to the development of insulin resistance [33].

Accumulation of an excess of lipids in the visceral fat compartment is closely associated to adipocyte hypertrophy in this tissue, which in turn leads to increased lipolysis [33]. The excess of lipids is shunted to the liver and skeletal muscle, which results in impaired insulin signalling within these tissues and consequent development of insulin resistance [34]. In this sense, adult AEA-treated mice during lactation should have an increased risk of ectopic lipid accumulation due to the elevated concentration of circulating NEFA, which may be originated from the higher content of visceral adipose tissue found in these animals. In addition, the higher accumulation of fat observed in AEA-treated mice may lead to a chronic inflammatory state which is associated to increased production of chemokines and pro-inflammatory cytokines such as tumor necrosis factor alpha (TNF-α), leading to higher circulating levels of these compounds. This condition may be contributing to the insulin resistance state observed in the present study. In this sense, it has been reported that elevated levels of TNF-α increase Ser307 phosphorylation of IRS-1 [35], therefore impairing the insulin signalling cascade. In addition, lipid accumulation leads to increased lipid oxidation, which results in enhanced oxidative stress, a condition known to contribute to a higher TNF-α secretion [36].

Recently, endocannabinoids have also been described as a class of lipid-derived mediators that are able to contribute to the pathogenesis of insulin resistance. It has been demonstrated that adipose tissue produce and is capable of secreting AEA and 2-AG [37-39]. Furthermore, it is likely that fat accumulation between muscle fibres, as a result of increased levels of plasmatic NEFA, could be accompanied by a higher local secretion of AEA. This secretion allows paracrine functions, leading to impaired insulin signalling. In this regard, it has been recently demonstrated that stimulation of skeletal muscle CB_1_R with AEA leads to impaired insulin-stimulated PKB/Akt (Ser^473^) phosphorylation and reduced insulin-stimulated glucose uptake [40], thus interfering with the signal transduction mechanisms involved in insulin actions. In addition, an AEA-independent mechanism leading to insulin resistance may involve intramyocellular fat accumulation through its consequent elevated availability of lipid metabolites such as ceramides and diacylglycerols, which affect insulin signalling [41].

Higher availability of AEA during lactation resulted in long term increased levels of CB_1_R in adipose tissue, which have been previously reported to be involved in lipogenesis. It was demonstrated that CB_1_R activation stimulates lipoprotein lipase (LPL) activity in cultured adipocytes from C57BL/6 N mice [14]. This enzyme hydrolyses circulating TG from VLDL and chylomicrons, facilitating the access of free fatty acids to adipocytes where they are accumulated again as TG. Thus, a higher LPL activity due to high levels of activated CB_1_R may lead to adipocyte hypertrophy and a higher release of free fatty acids to the circulation.

The two major endocannabinoids are originated from arachidonic acid (AA) containing phospholipids. This AA, together with linoleic acid, belongs to the n-6 family of essential fatty acids. Since linoleic and AA acids present in different tissues come from the diet, it is not unexpected that high and low intake of polyunsaturated fatty acids can influence levels of endocannabinoids. For example, feeding suckling piglets with a milk formula deficient in AA led to decreased levels of AEA and 2-AG in brain compared to piglets consuming sow milk [42]. In the same study, when mice were supplemented with AA for 58 days, brain levels of AEA increased by six-fold when compared to control animals [42]. Conversely, a higher intake of long-chain n-3 fatty acids can eventually lead to decreased levels of both 2-AG and AEA in brain and other tissues [43-45].

Although results in animal models may not be easily extrapolated to human beings, it is possible that the higher availability of n-6 fatty acids in Western diets could also contribute to a higher availability of endocannabinoids during fetal life and/or lactation, with important health consequences in adulthood, such as greater propensity to obesity.

## Conclusion

The results of the present study demonstrated that body fat accumulation, obesity/overweight, and its associated metabolic disturbances such as insulin resistance and a higher lipid profile, can be programmed at an early stage of life as a result of the activation of CB_1_ receptors with elevated doses of its agonist, the endocannabinoid AEA. Thus, we can suggest that treatment with AEA during lactation could be considered as a useful tool to study some long-term physiopathological manifestations of the metabolic syndrome.

## Abbreviations

CB1R, Type 1 cannabinoid receptor; AEA, Anandamide; NEFA, Non-sterified fatty acids; AA, Arachidonic acid; ITT, Insulin tolerance test; GTT, Glucose tolerance test; IAUC, Incremental area under the curve; DAUC, Decremental area under the curve; TG, Triglycerides.

## Competing interest

The authors declare that have no competing interests.

## Authors´ contributions

CAA and VAC performed all the experiments. MNLL and CAA participated in the design and the coordination of the study and in the drafting of the manuscript. All authors read and approved the final manuscript.
